# Effects of Olive Leaf Extract on Prevention of Molecular, Histopathological, and Enzymatic Changes in Chicken Carbon Tetrachloride-Induced Liver Damage

**DOI:** 10.31661/gmj.v8i0.1204

**Published:** 2019-07-07

**Authors:** Nazila Vahidi-Eyrisofla, Vida Hojati, Mohammad-Reza Yazdian, Morteza Zendehdel, Hooman Shajiee

**Affiliations:** ^1^Department of Biology, Damghan Branch, Islamic Azad University, Damghan, Iran; ^2^Department of Medical Science, Qom Branch, Islamic Azad University, Qom, Iran; ^3^Department of Basic Sciences, Faculty of Veterinary Medicine, University of Tehran, Tehran, Iran

**Keywords:** Olive, Liver, Carbon Tetrachloride, Chicken

## Abstract

**Background:**

Today, the use of additives such as antibiotics and growth hormones that increase production efficiency in breeding broiler chickens has become inevitable. However, the use of such additives and antibiotics associated with side effects such as liver damage. Oxidative stress occurs due to an imbalance between oxidants and antioxidants. Studies have shown that olive leaves have an antioxidant effect on free radicals. This study was to evaluate the possible effect of olive leaf extract on carbon tetrachloride (CCL4)-induced liver damage (molecular and tissue) and changes of enzymes in chickens.

**Materials and Methods:**

A total of 50 chickens were used and classified into5 groups. Treatment groups received 0.5, 1, and 1.5 mg/kg of the olive leaf extract from day 21 of the experiment. Two control groups—healthy and poisoned—did not receive any extract. On the day 35 of the experiment, 1cc of CCL4 was dissolved with olive oil and injected intraperitoneally into the experimental and poisoned control groups. Blood and liver tissue sampling were performed.

**Results:**

The histopathology results showed that at high doses of olive leaf extract, the cells and vessels were regularly curable, and sinusoids were healthy. The expression of B-cell lymphoma 2 (*BCL2*) increased, and that of BH3 interacting domain death agonist (*BID* )decreased. Enzymatic tests, including serum glutamic-oxaloacetic transaminase, serum glutamic-pyruvic transaminase, alkaline phosphatase, gamma-glutamyl transpeptidase, showed a reduction in *BID* expression in the experimental group compared with the control group(P<0.005).

**Conclusion:**

We concluded that olive leaf extract boosts the *BCL2* —an antiapoptotic gene—and reduces *BID* —an apoptosis gene—in the liver of chicken. It prevents the liver cells from disintegrating and destroys sinusoids and liver blood vessels. The high doses of the olive leaf extract caused liver resistance to CCL4 toxicity in chicken.

## Introduction

Today, the use of additives such as growth hormone, and elaborate on health incentives, which increase production efficiency, thereby leading to the increased production of breeding broiler chickens, has become inevitable. Antibiotics and probiotics can be used for the same reasons. Because antibiotic use can lead to side effects such as drug resistance, allergy, and liver damage, the importance of using herbs and herbal products for healthy food becomes more relevant [1-3]. The liver, which is the largest internal gland, can be damaged by drugs, chemicals, toxins, viruses, and parasites. In many cases, its pathogenesis is unclear. The role of the liver in detoxification is well known [4]. The tissue of the liver includes hepatocytes that are organized in the form of plates, the portal vein, and the central vein [5].BH3 interacting domain death agonist (*BID*) is an apoptotic pathway expression of protein through which other proteins interact, resulting in the formation of oligomeric pores in the mitochondrial membrane, thereby releasing the proapoptotic and cytochrome c supernatants [6]. Subsequently, the activation of caspase enzymes leads to cell death. Therefore, *BID* proteins that can affect the expression of this gene can increase or decrease the pathway for apoptosis [6]. B-cell lymphoma 2 (*BCL2*) family proteins maintain the integrity of the mitochondrial membrane and are composed of antiapoptotic and proapoptotic members. This family regulates the release of cytochrome c into the cytosol, and, once activated, regulates caspase-3 [7]. When there is an imbalance between antioxidants and oxidants, oxidative stress occurs [8, 9]. Studies have shown that due to a lower risk of side effects, there is a high tendency to use medicinal herbs to treat diseases that are caused by chemical drugs [10]. Due to the presence of antioxidants, some of the herbs or medicinal plants such as olive have a positive effect on the activities of various body systems [10]. Previous studies have shown that oleuropein and calcium elenolatein of the olive leaf possess antibiotic properties. They are effective against a wide range of bacteria as they destruct the bacterial cell walls and inhibit viral transcription as well [11, 12]. Also, the olive leaf extract is effective on a wide range of viruses, fungi, and yeasts [13, 14]. Studies have also indicated that oleuropein, 3, 4-dihydroxyphenylglycosides, esters, and flavonoids of the olive leaf have an antioxidant effect, and they act as free radical scavengers. Other elements in the olive leaves include selenium, zinc, chromium, iron, vitamin C, and beta-carotene [15-17]. Carbon tetrachloride (CCL4) is a solvent used in the chemical industry, and it is considered a toxic substance for the liver [18, 19]. During the metabolism of CCL4, 2toxic compounds trichloromethyl (CCl3) and trichloromethyl peroxy(Cl3COO) are produced, causing liver diseases such as cirrhosis and necrosis [18].This study showed that the olive leaf extract is effective in pathological and physiological processes in the liver. The study was designed to evaluate the possible effect of the olive leaf extract on CCL4-induced liver damage (molecular, enzymatic, and tissue) in chicken.

## Materials and Methods

### Materials

The starter diet, the growth diet, and vaccines were bought from Minoo Sabah Golestan and Pasouk, Iran. The experimental and staining kits were bought from Hiva, Iran.

### Animals

In the present study, 50 one-day-old chickens (Ross 308) were categorized into 5 groups (n=10 per group). During the experiment, the light was supplied to the chickens 24 hours a day, with free water and food. The temperature in the first week of breeding was about 30°C and gradually decreased every 3 days by 1°C to reach 23°Cto 24°C. The vaccination program was conducted in accordance with the global standards table. Two diets (starter and growth) were used for all experimental treatments based on the recommendations of the National Research Council (NRC, 1994). The starter diet (from days 1 to 21) contained 2900 kcal of energy and 20.84% crude protein, whereas the growth diet (from day 22 to the end of the course) contained 3000 kcal of energy and 18.75% crude protein.

### Experimental Groups

With the help of the gavage syringe, the olive leaf extract was injected in the chickens of the treatment group for 14 days from day 21 of the experiment. Three experimental groups received 0.5, 1, and 1.5 mg/kg body weight of the aqueous extract of olive leaves, which was mixed with 2cc distilled water; the two control groups (healthy and poisoned) received no any extract. On the 35th day of the experiment, 1 cc of CCL4 was dissolved with olive oil (1:1 v/v) and injected intraperitoneally into the experimental and poisoned control groups. Blood samples were obtained from the wing vein of chickens, and then blood sampling was done at 1, 6, 12, and 24 hours after injection. Liver tissue sampling was done once chickens were unconscious.

### Histopathology

The tissue samples were fixed in 10% formalin and then molded in paraffin. Slices of paraffin blocks, sections measured 3 to 5 µm, were prepared using a microtome (Sleemainz, Model: Cut 4055, Sakura, Japan). Thereafter, the paraffin slices were placed on the glass slides, followed by paraffin dehydration, dewatering, and finally stained with hematoxylin and eosin (H&E, Baditeb, Iran).

### Determination of Liver Enzymes and Bilirubin in Serum

Samples were centrifuged at 3500 rpm for 5 minutes, and the serum was isolated. The concentration of liver enzymes, including serum glutamic-oxaloacetic transaminase (SGOT), serum glutamic-pyruvic transaminase (SGPT), alkaline phosphatase (ALKP), gamma-glutamyl transpeptidase (GGT), total bilirubin (BILI.T), and direct bilirubin (BILI.D), was measured using the spectrophotometric method and with the help of theBT1500 (Biotecnica Instruments SpA, Rome, Italy). The manufacturer’s recommendations were followed while using the device.

### BID and BCL2Genes Expression

For molecular testing, 25mg of liver tissue was isolated from each group and homogenized in liquid nitrogen. Then, the whole tissue was extracted using GeneAll (Pishgam, Iran) in accordance with the protocol. Using a spectrophotometer, the total purity of the ribonucleic acid and the absorbance of the wavelengths from 260 to 280 nm were measured. In this study, specimens with 260:280 nanometers optical density (OD) were used between 1.8 and 2.2 for complementary DNA(cDNA) synthesis. Then, the amount of 1 µg of RNA was converted to the cDNA using the HyperScriptTM RT premix kit with Random Hexamer (Pishgam, Iran), following the cDNA protocol. For the synthesis of cDNA, the tissue sample was heated at 25°C for 10 minutes, followed by incubation at 55°C for 55 minutes and 95°C for 5 minutes. Following the synthesis of cDNA, the polymerase chain reaction was initiated. The real-time polymerase chain reaction (SYBR Green, Yekta tajhiz, Iran) was performed on a Corbet machine (6000 Rotor Gen, Corbett, Germany). The primers of *BID, BCL2,* and *GAPDH* genes (reference genes) are given in Table-1. The temperature schedule of the machine was adjusted in 3 steps. The first stage leads to the denaturation of the cDNA molecules (95°C for 12 min). The second stage was 93°C for 20 seconds for the spell, 35 seconds at different connection temperatures (Table-1) for annealing and 25 seconds at 72°C for extension in 37 successive sequences. To confirm the reproduction of each component, the gene and the absence of nonspecific products and primer pairing in the early studies of electrophoresis with 2% agarose gel and routine of the melting curve were used. To plot the melting curve, the temperature at each repetition was between 50°Cand 99°C, and at every 5 seconds, it was increased by1°C.

## Results

### Effect of Olive Leaf Extract on Liver Tissue

**Figure 1:**
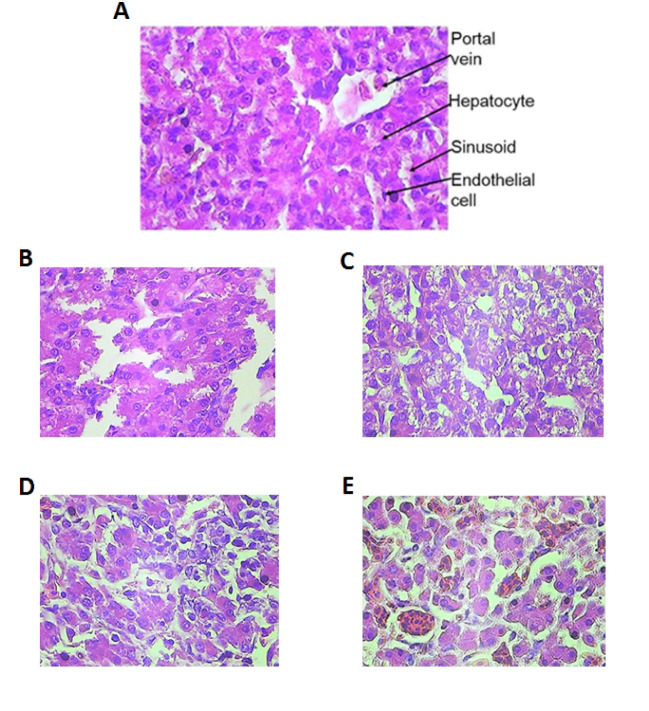
Microscopic images of the liver (×400, H&E staining). **A:** In the healthy control group, the rows of normal liver cells, bile ducts, sinusoids, and blood vessels. **B:** In olive leaf extract group (0.5 mg/kg), cell degradation and disintegration of the hepatocyte cell line and the destruction of sinusoids were seen. **C:** A degree of destruction between the cells were observed in 1 mg/kg group. **D:** Treatment with 1.5 mg/kg of olive leaf extract showed normal cells, bile ducts, and blood vessels.**E:** The most hepatic cell dislocation, as well as the presence of cells in the bile duct, and sinusoidal degeneration were observed in the toxic control group.

The histological results showed that the healthy control group, which did not receive any extracts, had healthy liver cells, and their bile ducts and blood vessels were normal (Figure-1A). In the poisoned control group, which did not receive any extracts but had CCL4 injections, cellular infiltration and the presence of the cell in bile ducts were detected (Figure-1B). In groups that received the olive leaf extract of 0.5 mg/kg, cellular and sinusoidal degenerations were quite apparent (Figure-1C). However, in the groups that received the dose of 1 mg/kg, the degeneration was less than those that received the dose of 0.5mg/kg (Figure-1D). In the groups that received the dose of 1.5 mg/kg, the cells and veins were curable, and sinusoids were healthy (Figure-1E).

### Effect of Olive Leaf Extract on Liver Enzymes, BILI.T, and BILI.D

**Figure 2:**
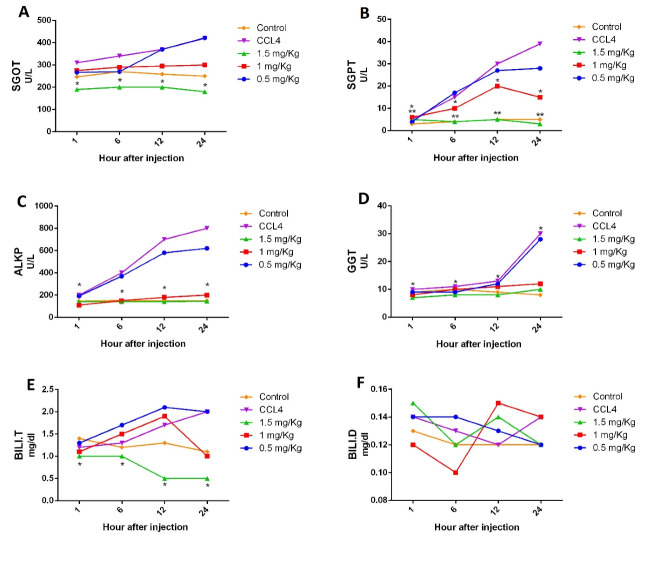
**A:** The SGOT tests showed high dose of olive leafs have prevent the liver damage in comparison with CCL4 group (*P <0.05). **B:** Olive leafs in two doses (1 and 1.5 mg/kg) can prevent the liver damage in compare with CCL4 group (*P<0.05 and **P<0.005). **C:** The ALKP test shows olive leaf in 1 and 1.5 mg/kg dose can prevent the liver damage in comparison with CCL4 group (*P<0.005). **D:** The GGT test shows olive leaf in 1 and 1.5 dose can prevent the liver damage in comparison with CCL4 group (*P<0.05). **E:** BILI.T test shows 1.5 mg/kg dose of the olive leaf can prevent the liver damage in compare with CCL4 group (*P<0.005). **F:** BILI.D test revealed there is no significant difference in comparison with poison control group.

The maximum effect of CCL4 observed after 24 hours of injection, which is a good time to compare its effects on various groups. From being impaired in all of these charts, the toxic group showed a rise in liver enzymes, resulting in liver damage. As shown in Figure-2A, in the SGOT test, a high dose of the olive leaf extract (1.5 mg/kg) prevented liver damage (P=0.044). In the SGPT test (Figure-2B), 2 doses of the olive leaf extract (1 and 1.5 mg/kg) could prevent liver damage (P=0.028 and P=0.001, respectively). Figure-2C shows that in the ALKP test, the doses of the 1 and 1.5 mg/kg olive leaf extract could prevent liver damage (P=0.003). Figure-2 Dshows the GGT test in which the 1 and 1.5 mg/kg doses of the olive leaf extract could prevent liver damage (P=0.033,and P=0.014, respectively). Figure-2E shows the BILI.T test in which the 1.5 mg/kg dose of the olive leaf extract could prevent liver damage (P=0.003). Figure-2F shows the BILI.D test where no significant difference in comparison with the poison control group was found. In all experiments, the 0.5 mg/kg dose of the olive leaf extract showed no significant difference. All the 5 groups, including the poisoned and healthy control groups, received the 0.5, 1, and 1.5 mg/kg doses of the olive leaf extract. The patterns show the increase in the levels of serum after 1, 6, 12, and 24 hours of CCL4 injection.

### Effect of Olive Leaf Extract on BID and BCL2Genes Expression

**Figure 3:**
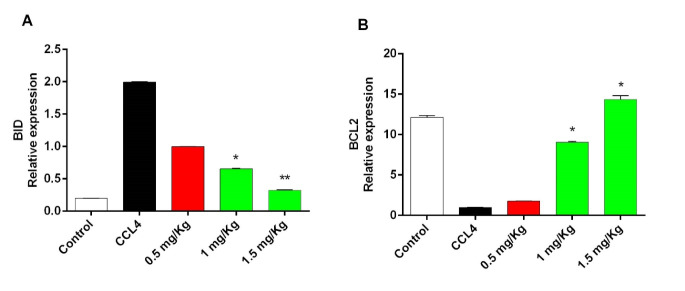
The BID expressions decrease with increasing does of olive leaf extract, in 1 and 1.5 mg/kg;** B:** The BCL2 expressions increase with increasing does of olive leaf extract, in 1 and 1.5 mg/kg. *P<0.05, **P<0.005 vs. CCL4 group.

In the experimental groups, with an increase in the dose of the olive leaf extract, the antiapoptotic gene expression level of *BCL2* increased in comparison with that of the control group, and, on the other hand, the *BID* expression in the treatment groups decreased with a rise in the dose of the olive leaf extract (P=0.001, Figure-3). In the present study, increasing the expression of the *BCL2* gene and reducing the expression of *BID* gene after the injection of different doses of the olive leaf extract, the mitochondrial membrane instability decreased, and thus its permeability decreased with respect to cytochrome C. Therefore, it can be concluded that olive leaf extract improves the mitochondrial membrane and thus prevents the development of apoptosis through the internal pathway in the liver of the chicken. However, further studies are needed to find other paths in which olive leaves interfere.

## Discussion

A component of the liver profile is aspartate aminotransferase that flows into the blood when the liver gets damaged [19]. Alanine aminotransferase helps the liver convert food into energy. When the liver is irritated or injured, it leaks into blood [20], and increased levels of alkaline phosphatase indicate the problem with the gallbladder, bones, or liver [21]. The enzyme that is found in the kidney, pancreas, other tissues, and mainly in the liver are GGT. In general, the increased level of this enzyme indicates liver diseases [22]. The breakdown of red blood cells leads to the formation of bilirubin, which travels to the liver. The excess of bilirubin leads to the inflammation or obstruction of the liver [23]. Increased liver enzymes in blood serum are one of the main indicators of liver damage due to their toxic effect [24]. During CCL4 poisoning, fat from the surrounding adipose tissue is transmitted to the liver and kidneys, leading to fat accumulation in them, and ultimately resulting in tissue damage [25]. CCL4 is converted to the CCl3 (a highly reactive free radical) by an enzymatic system NADPH cytochrome P-450 present in the endoplasmic reticulum (ER) of the liver cells. Then it reacts with oxygen and converts to Cl3COO and is attacked by the endoplasmic reticulum membrane, causing lipid peroxidation, cell calcium loss, decreased protein synthesis, increased liver enzymes, and ultimately leads to the degeneration of liver cells [26, 27].Dietary supplementation with olive oil in the liver of high-fat-diet (HFD)-fed mice may prevent oxidative stress, resulting in the reduction of biosynthesis and accretion of ω-3 long-chain polyunsaturated fatty acids [28]. Polyphenol-rich virgin olive oil or olive oil without polyphenols in HFD-fed rats limits the HFD-induced resistance of insulin, inflammation, and hepatic oxidative stress, and thus prevents nonalcoholic fatty liver disease progression [29]. Some doses of olive are associated with increased liver antioxidant system parameters;2 weeks of oral pretreatment with different doses of olive decreased renal injury caused by renal ischemia-reperfusion and increased renal catalase and glutathione peroxidase activities [30]. The most relevant molecular effects of olive involved in the prevention of liver damage are the activation of the nuclear transcription factor (erythroid-derived 2)-like 2, the inactivation of the nuclear transcription factor NF-κB (preventing the cellular inflammatory response), and the inhibition of the protein kinase RNA (PKR)-like ER kinase pathway [31]. A study showed that the combination of PGE1 and iodized olive oil prevented the development of liver fibrosis following transarterial chemoembolization [32]. The extract of *Salviacryptantha* supplementation had a protective effect against CCL4-induced liver damage [33]. The chickens fed with polyphenols polyphenolic powder from olive showed reduced oxidative stress-induced damage [34]. The olive fruit phenolic compounds can be used as protective compounds against liver and renal toxicity induced by deltamethrin [35]. The administration of the *Teucriumpolium* Lextract to mice significantly reduced the level of liver enzymes compared with that of the CCL4 poisoned group [36]. The use of the Turkish folk ethanolic extract in mice who were poisoned with CCL4 prevented the increase of alanine transaminase and aspartate transaminase liver enzymes in blood serum [37]. Pharmacological properties of a major phenolic compound oleuropein include anticancer activities, antimicrobial activity, antioxidant, anti-inflammatory, antiatherogenic, antiviral activity, and hypolipidemic and hypoglycemic effects [38]. C11H14O6 and C25H32O13 are the glucosylated components that are produced during the maturation of olive from oleuropein [39].In an experiment of cell culture, 3-, 4-dihydroxyl-phenyl and p-hydroxyl-phenyl ethanol, which come from olive oil phenolic, were shown to possess antioxidant properties, as well as ortho-dihydroxy property that is essential for molecules [40].

## Conclusion

Overall, the results showed that high doses of the olive leaf extract cause liver resistance to CCL4toxicity in chickens. The olive leaf extract boosts the antiapoptotic expression of the *BCL2* gene, reduces *BID* gene apoptosis, prevents the liver cells from being disintegrated, destroys sinusoids and liver blood vessels, and prevents the increase of liver enzymes. The olive extract prevents oxidative stress because of its antioxidant properties, and it also prevents apoptosis through the internal pathway of the chicken liver by improving the mitochondrial membrane. However, further studies are needed to find other paths in which olive leafs interfere. It is recommended to prevent liver damage by using the extract of olive leaves in the diet of chickens. The reason for this is that it is easily available at low cost. In future research, the effects of olive leaves on the liver will be addressed.

## Conflict of Interest

None declared.

**Table 1:** Sequence, Size, and Temperature of Used Binding Primers

**Name**	**Primer sequence (5’-3’)**	**Size (bp)**	**Annealing (°C)**
***BID***	F:GCTATGAGTTACTGCGTTCG R: CTCTTTGAACTCACAGCCAG	182	57
***BCL2***	F: ATTTTATTACCGTTGGCTG R: CTGCGAGAGTTTATAGTGGA	167	56
***GAPDH***	F: GCAGGAACACTATAAAGGCG R: CCCTTGAAGTGTCCGTGTG	189	55

## References

[1] Vahidi eyrisofla N, Ahmadifar M, Eini AM, Kalami A (December 29, 2014). The Study of Levofloxacin Effects on Liver Tissue in Wistar Rat. Journal of Liver.

[2] Amamou F, Nemmiche S, Meziane R.k, Didi A, Yazit S-M, Chabane-Sari D. Protective effect of olive oil and colocynth oil against cadmium-induced oxidative stress in the liver of Wistar rats. Food and Chemical Toxicology. 2015 Apr; 78:177-84. 10.1016/j.fct.2015.01.001 25617810

[3] Androli T, Carpenter C, Griggs R, Benjamin I. Diseases of the Liver and Biliary System. Cecil’s Essentials of Medicine. 29th March 2010. EBook ISBN: 9781455753543.

[4] Lee K, Everts WH, Kappert HJ, Yeom KH, Beynen AC (1 December 2003). Dietary carvacrol lowers body weight gain but improves feed conversion in female broiler chickens. The Journal of Applied Poultry Research.

[5] Sherlock S, Dooley J (2003). Discases of the liver and bilary system. Gut.

[6] Chen B, Peng X, Pentassuglia L, Lim CC, Sawyer DB (2007). Molecular and cellular mechanisms of anthracyclinecardiotoxicity. Cardiovasc Toxicol.

[7] Nicholson. DW, Thornberry. NA. 2003. Apoptosis. Life and death decisions. 10.1126/science.1081274 12522239

[8] Dalton TP, Shertzer HG, Puga A (April 1999). Regulation of gene expression by reactive oxygen. Annual Review of Pharmacology and Toxicology.

[9] Scandalios JG. Genomic responses to oxidative stress. Encyclopedia of Molecular Cell Biology and Molecular Medicine. 15 September 2006.

[10] Cotelle N, LucBernier J, PierreCatteau J, Pommery J, ClaudeWallet J, M.Gaydou E (1996). Antioxidant properties of hydroxy-flavones. Free Radical Biology and Medicine.

[11] Wilms LC, Hollman PC, Boots AW, Kleinjans JC (2005 Apr 4). Protection by quercetin and quercetin-rich fruit juice against induction of oxidative DNA damage and formation of BPDE-DNA adducts in human lymphocytes. Mutat Res.

[12] Polzonetti V, Egidi D, Vita A (v2004). Involvement of oleuropein in (some) digestive metabolic pathways. Food Chemistry.

[13] Bisignano G, Tomaino A, Lo Cascio R, Crisafi G, Eccella N, Saija A. In-vitro antimicrobial activity of oleuropein and hydroxytyrosol. The Journal of Pharmacy and Pharmacology. 18 February 2010. 10.1211/0022357991773258 10504039

[14] Paul S, Nash DC (July 14,1997). Olive leaf extract regains interest as a superb anti- microbial agent. Dynamic Chiropractic.

[15] Benavente-Garcia O, Castillo J, Lorente J, Ortuno A, Del Rio J (March 2000). Antioxidant activity of phenolics extracted from oleaeuropaea L leaves. Food Chemistry.

[16] AngelRincón M, Valenzuela R, CHernandez-Rodas M, Marambio M, Espinosa A, Maye Sr, Romero N, Barrera C, ValenzuelaLuis A, Videla A (2016 Nov-Dec). Supplementation with antioxidant-rich extra virgin olive oil prevents hepatic oxidative stress and reduction of desaturation capacity in mice fed a high-fat diet: Effects on fatty acid composition in liver and extrahepatic tissues. Nutrition.

[17] Lama A, Pirozzi C, Mollica MP, Trinchese G, Di Guida F, Cavaliere G, Calignano A, Mattace Raso G, Berni Canani R, Meli R (017 Mar). Polyphenol-rich virgin olive oil reduces insulin resistance and liver inflammation and improves mitochondrial dysfunction in high-fat diet fed rats. Molecular nutrition.

[18] Janbaz KH, Saeed S, Gilani AH (2002 Dec). Protective effect of rutin on Paracetamol and CCl4- induced hepatotoxicity in rodents. Fitoterapia.

[19] Suzanne Falck, MD. August 21, 2017. Medically reviewed. https://www.healthline.com/health/sgot-test.

[20] Larissa Hirsch., MD. November 2017. Medically reviewed. http://kidshealth.org/en/parents/test-alt.html.

[21] Stacy Sampson, DO. August 1, 2017.Medically reviewe. https://www.healthline.com/health/alp.

[22] Rugheed Ghadban, MD. Dec 11, 2013. Medically reviewe. https://emedicine.medscape.com/article/2087891-overview.

[23] Medically reviewed by Stacy R. Sampson, DO on March 3, 2017 — Written by Christine Case-Lo. https://www.healthline.com/health/bilirubin-blood.

[24] Hetrog MGL, Hollmann PCH (1996 Feb). Potential health effects of the dietary flavonol quercetin. European Journal of Clinical Nutrition.

[25] Yalcin A, Yumrutas O, Kuloglu T, Elibol E, Parlar A, Yilmaz I (2017). Hepatoprotective properties for Salvia cryptantha extract on carbon tetrachloride-induced liver injury. Cell Mol Biol (Noisy-le-grand).

[26] Boll M, Weber LW, Becker E, Stampfl A (2001 Jan-Feb). Pathogenesis of carbon tetrachloride in hepatocyte injury Bioactivation of CCl4 by cytochrome P450 and effects on lipid homeostasis. Z Naturforsch.

[27] Clawson GA (1989). Mechanism of carbon tetrachloride hepatotoxicity. Pathology and Immunopathology Research.

[28] Fallah Huseini h, ZareeiMahmoudabady A, Ziai SA, Mehrazma M, Alavian SM, Mehdizadeh M (2011). The effects of CynarascolymusL Leaf and Cichoriumintybus L root extracts on carbon tetrachloride induced liver toxicity in rats. Medicinal Plants.

[29] Amiot M, Fleuriet A, Macheix J (1989). Accumulation of oleuropein derivatives during olive maturation. Phytochemistry.

[30] Jafarpour L, Rasoulian B, Tavafi M, Rafighdoost H, Mahmod Mi, Rashidipour M, Ahmadvand H (2016). Pretreatment with Olive Leaf Extract Improves Renal and Liver Antioxidant Systems Following Renal Ischemia-Reperfusion Injury in Rats. Medicines Journal December.

[31] Soto-Alarcon SA, Valenzuela R, Valenzuela A, Videla LA (2018). Liver Protective Effects of Extra Virgin Olive Oil: Interaction between Its Chemical Composition and the Cell-signaling Pathways Involved in Protection Endocrine. Metabolic & Immune Disorders-Drug Targets.

[32] Jin S, Cao H, Wang K, Li Y, Bai B (2015 Jun). Preventative effects of prostaglandin E1 in combination with iodized olive oil on liver fibrosis after transcatheter arterial chemoembolization in a rabbit model of CCl4-induced liver fibrosis. Canadian journal o physiology and pharmacology.

[33] Alper Yalcin, Onder Yumrutas, Tuncay Kuloglu, Ebru Elibol, Ali Parlar, İsmet Yilmaz, Mustafa Pehlivan, Mevlut Dogukan, Fatih Uckardes, Hasan Aydin, Ahmet Turk, Oznur Uludag, İbrahim Sahin, Kader Ugur, Suleyman Aydin. 2016. Hepatoprotective properties for Salvia cryptantha extract on carbon tetrachloride-induced liver injury. Cellular and molecular biology. 10.14715/cmb/2017.63.12.13 29307343

[34] Papadopoulou. A. Petrotos K, Stagos D., Gerasopoulos K., Maimaris A., Makris H., Kafantaris I., Makri S., Kerasioti E., Halabalaki M., Brieudes V., Ntasi G., Kokkas S.,.Tzimas P, Goulas P., Zakharenko A.M., Golokhvast K.S., Tsatsakis A., Kouretas D.. Enhancement of Antioxidant Mechanisms and Reduction of Oxidative Stress in Chickens after the Administration of Drinking Water Enriched with Polyphenolic Powder from Olive Mill Waste Waters. Oxidative Medicine and Cellular Longevity. Oxidative Medic 10.1155/2017/8273160 PMC5613686 29138680

[35] Maalej A, Mahmoudi A, Bouallagui Z, Fki I, Marrekchi R, Sayadi S (2017 Aug). Olive phenolic compounds attenuate deltamethrin-induced liver and kidney toxicity through regulating oxidative stress, inflammation and apoptosis. Food Chem Toxicol.

[36] Panovska TK, Kulevanova S, Gjorgoski I, Bogdanova M, Petrushevska G (16 May2007). Hepatoprotective effect of the ethyl acetate extract of TeucriumpoliumL Againstcarbontetrachloride-induced hepatic injury in rats. ActaPharmaceutica.

[37] Aktay G, Deliorman D, Ergun F, Yesiladan E, Cevik C (November 2000). Hepatoprotective effects of Torkish folk remedies on experimental liver injury. Journal of Ethnopharmacology.

[38] OMAR SH (2010). Oleuropein in Olive and its Pharmacological Effects pharmaceutica. Sci.Pharm.

[39] Campbell, T. W., and Coles, E. H. 1986. Avian Clinical Pathology. In: Veterinary Clinical Pathology. Edited by E.H. Coles. 4th Ed. W. B. Saunders Co. Philadelfia.

[40] Manna C, Galletti P, Cucciolla V, Moltedo O, Leone A, Zappia V (Feb). The Protective Effect of the Olive Oil Polyphenol (3, 4-Dihydroxyphenyl) - ethanol Counteracts Reactive Oxygen Metabolite–Induced Cytotoxicity in Caco-2 Cells. JN the journal of nutrition 1997.

